# Small sharp exostosis tip in solitary osteochondroma causing intermittent knee pain due to pseudoaneurysm

**DOI:** 10.1186/1756-0500-6-142

**Published:** 2013-04-10

**Authors:** Wiebke K Guder, Arne Streitbürger, Georg Gosheger, Michael Köhler, Dagmar Bachhuber, Marcel-Philipp Henrichs, Jendrik Hardes

**Affiliations:** 1 Department of Orthopedics and Tumor Orthopedics, University Hospital Muenster, Albert-Schweitzer-Campus 1, Building A1, 48149, Muenster, Germany; 2 Department of Clinical Radiology, University Hospital Muenster, Albert-Schweitzer-Campus 1, Building A1, 48149, Muenster, Germany; 3 Department of Vascular and Endovascular Surgery, University Hospital Muenster, Albert-Schweitzer-Campus 1, Building A1, 48149, Muenster, Germany

**Keywords:** Osteochondroma, Pseudoaneurysm, Complication

## Abstract

**Background:**

Complications of solitary or multiple osteochondromas are rare but have been reported in recent literature. Most reported complications arose in patients with multiple and/or sizable osteochondromas.

**Case presentation:**

A 22-year-old, female, Caucasian patient with obesity presented with intermittent knee pain and hematoma of the right calf. The MRI depicted a small, sharp exostosis tip of the dorsal distal femur with a surrounding soft-tissue mass. After profuse bleeding occurred during biopsy of the soft tissue mass, angiography revealed a pseudoaneurysm of the right popliteal artery. In a second-stage surgery the exostosis tip and pseudoaneurysm were resected.

**Conclusion:**

Complications can also arise in small, seemingly harmless osteochondromas. Surgical resection should be considered as a preventive measure when exostoses form sharp tips close to neurovascular structures regardless of total osteochondroma size.

## Background

Complications arising from solitary or multiple osteochondromas are rare but have been reported in recent literature. Most often they are described to occur in patients with multiple or sizeable exostoses [1-3]. In appendicular locations complications such as development of pseudoaneurysms [4], ruptures of pseudoaneurysms [5], thrombosis [6] and ischemia [7] have been reported. Spinal cord compression [8], hemothorax [1] and digestive obstruction [9] have occurred in locations of the torso. Clinical symptoms are often unspecific or occur after traumatic stress in the affected location [10]. Persistent pain, pressure on adjacent structures (such as nerves), limitations of joint movement and suspected malignant transformation are common indications for surgical resection of an exostosis [3].

We present the case of a small solitary exostosis with a sharp tip that caused a pseudoaneurysm to show that severe complications can occur regardless of osteochondroma size. Thus, we propose that surgical resection is indicated as a preventive or therapeutic strategy in selected cases of small osteochondromas depending on their shape and site.

## Case presentation

In September 2011, a 22-year-old, female, Caucasian patient with obesity (BMI 38,9; height 169 cm, weight 111 kg) first presented to our outpatient clinic with an unclear lesion of the right dorsal distal femur with a surrounding soft tissue mass. Patient history was empty except for intermittent arterial hypertension and having been treated for an inguinal hernia in childhood. The patient recounted that she had presented to her general physician with an onset of knee pain on the right side. She then received conservative treatment for the suspected diagnosis of a burst baker-cyst. In the course of treatment, plain x-rays and MRI imaging of the knee were obtained due to persistent pain.

**Figure 1:**
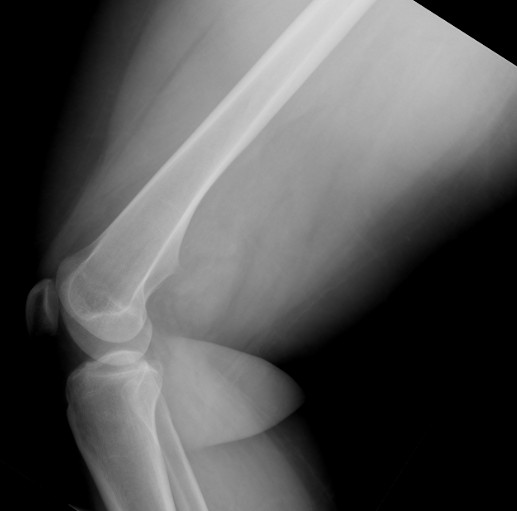
**X-ray of the right distal femur (lateral view).** The x-ray shows the outline of a broad-based osteochondroma with a sharp tip in a dorsal location.

**Figure 2:**
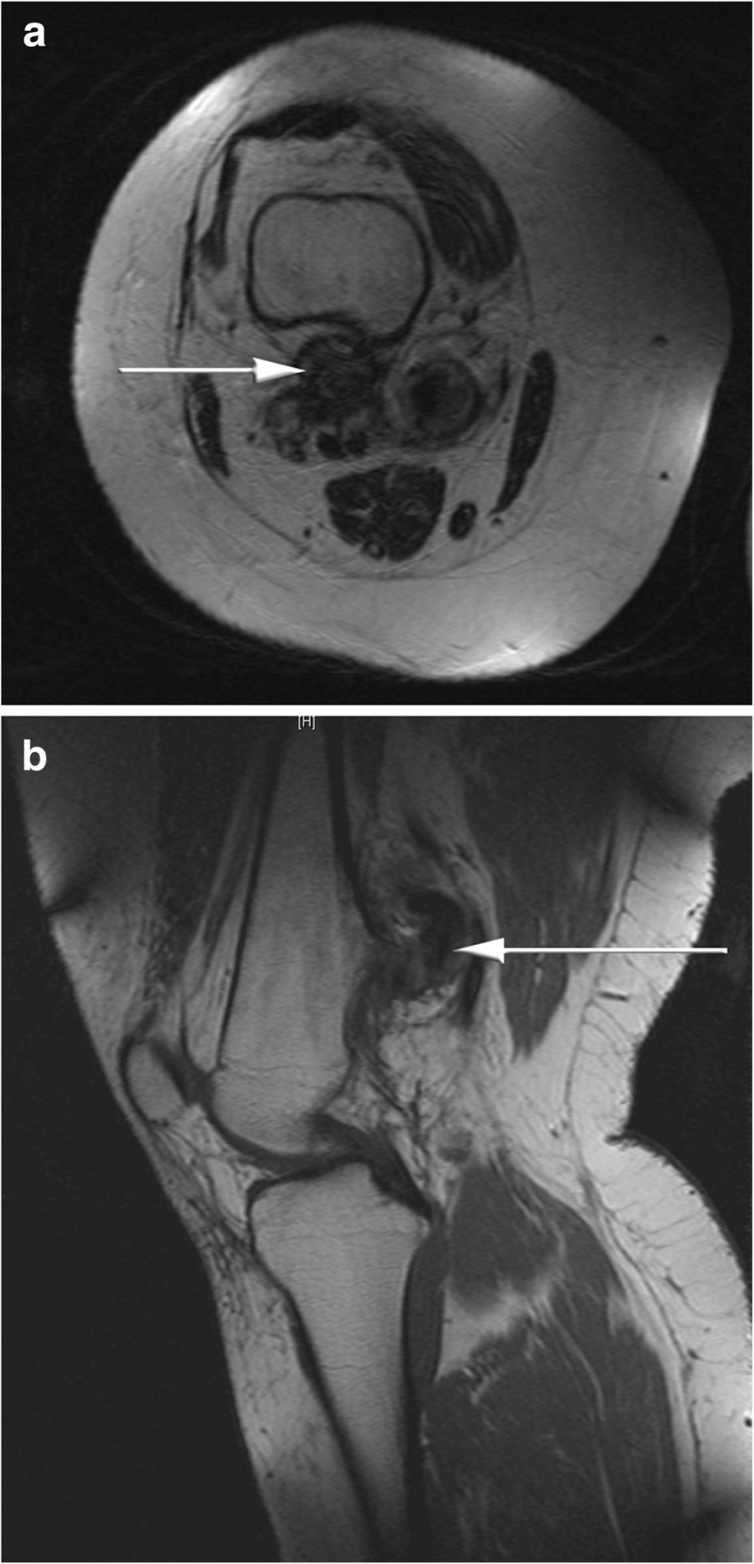
**MRI of the right distal femur. a**. MRI (T2-TSE-TRA-512) depicting a soft tissue component being pierced by sharp exostosis tip (axial view); white arrow points to soft tissue mass. **b**. MRI (T1-SE-SAG-512) depicting a soft tissue component around sharp exostosis tip (sagittal view); white arrow points to soft tissue mass.

At first presentation at our outpatient clinic, the patient had a hematoma of the right calf. Due to pyknic habitus, the range of motion was limited and the lesion couldn’t be palpated in the right popliteal fossa. The peripheral perfusion was intact; there were no sensory or motoric deficits. The lateral radiograph of the right distal femur depicted what seemed like a broad-based exostosis with a small tip extending into the popliteal fossa (Figure 1). The MRI also depicted what appeared to be an exostosis tip and a surrounding soft tissue mass (Figure 2a, 2b) bordering on the popliteal vessels. Multiple exostoses were excluded by clinical examination.

To exclude a secondary chondrosarcoma or other malignancies the patient was scheduled to undergo an incisional biopsy. Surgery was performed using a lateral approach and a longitudinal incision in the right, distal femur.

Intraoperatively, digital palpation of the soft tissue mass showed an arterial pulsation and profuse bleeding occurred upon palpation. A tourniquet was applied in the proximal thigh to achieve haemostasis and the leakage was sutured by a vascular surgeon.

**Figure 3:**
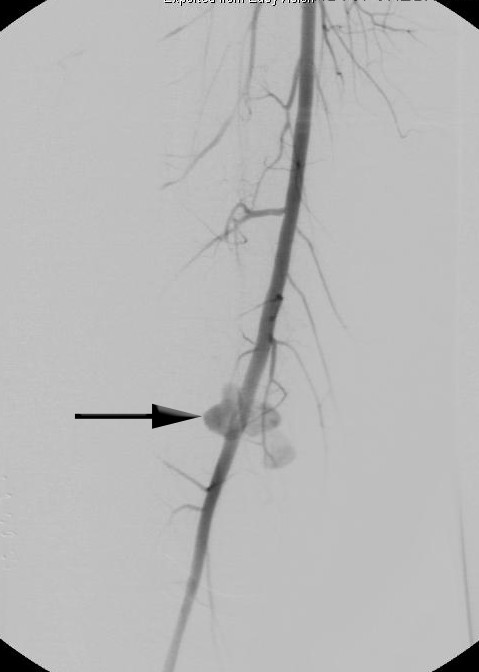
**Angiography of the right popliteal artery.** The picture depicts a polycyclic, popliteal aneurysm with smooth margins in P1 segment without active signs of leakage outside the aneurysm cavity; black arrow points to popliteal aneurysm.

To verify the intraoperatively suspected diagnosis of pseudoaneurysm, the patient underwent a diagnostic angiography after surgery was concluded. The angiography depicted a polycyclic popliteal aneurysm with smooth margins in the P1 segment. There were no signs of active leakage outside of the aneurysm formation. Muscular branches of the superior femoral artery were in close location to the aneurysm cavity but showed no communication. The diagnosis of aneurysm spurium was confirmed (Figure 3).

**Figure 4:**
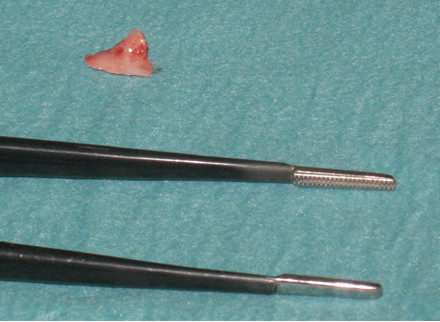
Resected exostosis tip responsible for punctured hole in popliteal artery.

**Figure 5:**
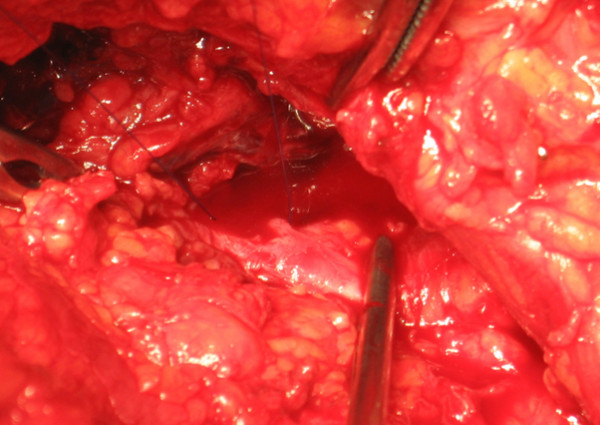
Suture of the 2 mm-hole in the right femoral artery.

After consultation with the vascular surgeons, another interventional surgical approach was planned. In a second surgery, the sharp exostosis tip and aneurysm spurium were resected. (Figures 4– 5) The exostosis tip had pierced a 2 mm hole (in diameter) into the popliteal artery that was also sutured during surgery.

Histological examination confirmed a fresh thrombus with parts of necrotic and haemorrhaged adipocytic tissue.

**Figure 6:**
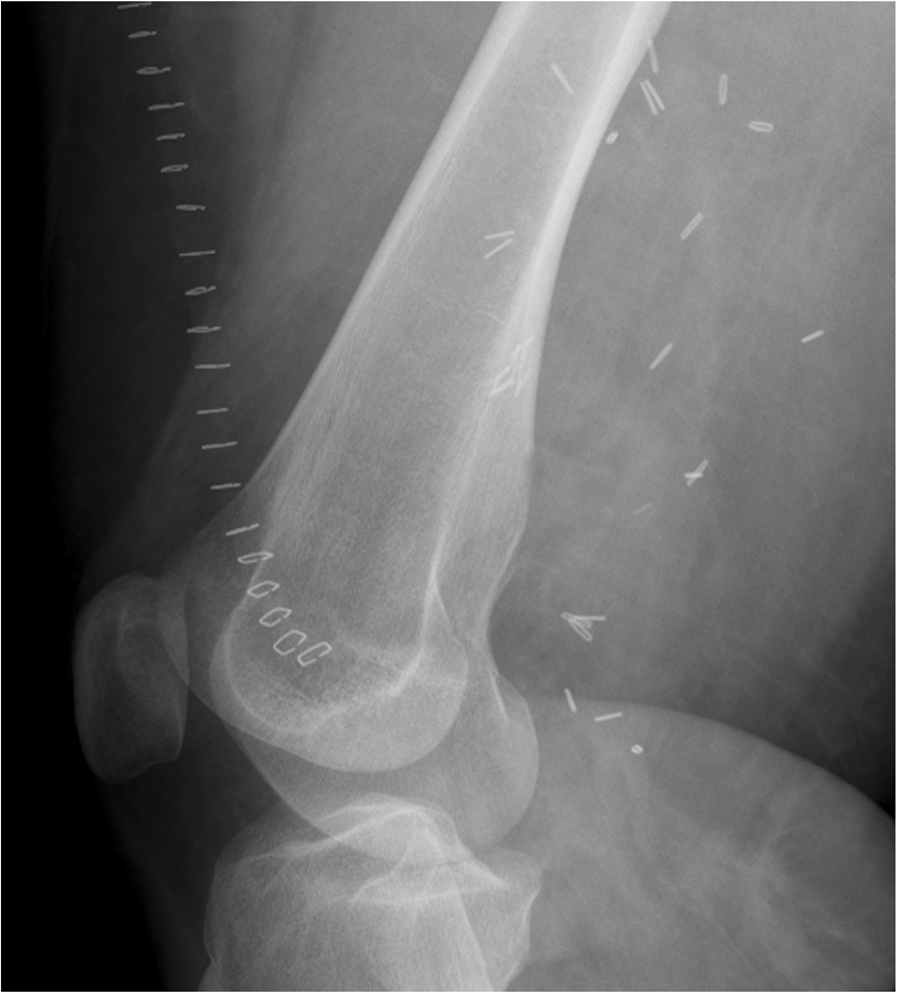
Plain x-ray after resection of the exostosis tip (lateral view).

Post-operative follow-up on the hospital ward showed good wound-healing and mobilisation of the patient who was discharged nine days after interventional surgery. After vascular surgery the patient was recommended to take acetylsalicylic acid 100 mg 1-0-0 for 6 months. The postoperative X-ray shows the remaining broad-based exostosis without the sharp tip (Figure 6). One year after surgery, the patient is pain free and doesn’t suffer from limitations in terms of joint movement of the persistent broad base of the exostosis.

## Discussion

This case report presents a rare vascular complication caused by a solitary, broad-based osteochondroma with a small, sharp exostosis tip. The patient history of knee pain combined with the onset of a hematoma should lead to doubts regarding the initially suspected diagnosis of a ruptured baker cyst. In patients with unclear pain and hematoma as well as a suspected exostosis with soft tissue mass in MRI imaging, we recommend further MRI imaging with contrast agent in accordance to accepted guidelines for the diagnosis of a soft tissue mass [11] - and a CT angiography if the soft tissue mass is located adjacent to blood vessels - to be obtained before biopsy to minimize the risk of surgical complications or false treatment.

Therefore, in exostoses presenting with unclear knee pain we recommend taking a thorough patient history and careful review of the available radiographs and MRIs. Complications in patients with exostoses are most often reported in cases of hereditary multiple or sizeable exostoses [5,12] compared to solitary ones [7,13]. We think it is important to be aware that seemingly harmless, solitary exostoses with only small tips may cause major vascular complications as well and need to be resected to prevent vascular complications in an optimized operative setting.

## Conclusion

Most existing case reports refer to complications arising from sizable osteochondromas in patients with multiple hereditary osteochondromas. Common reasons for resections of osteochondromas are compression symptoms, pain, limited range of motion or suspected malignancy.

We think it is important to consider that sharp tips in exostoses regardless of size may also be cause to complications and resection should be considered as a preventive measure or after complications like the formation of a pseudoaneurysm have occurred.

## Consent

Written informed consent was obtained from the patient for publication of this Case report and any accompanying images. A copy of the written consent is available for review by the Editor of this journal.

## Competing interests

The authors declare that they have no competing interests.

## Authors’ contributions

WKG review of current literature conception and writing of manuscript AS critical review of manuscript from orthopedic point of view, GG critical review of manuscript from orthopedic point of view, MK critical review of manuscript from a radiologic point of view, DB critical review of manuscript concerning vascular surgery, MPH review of current literature, draft of manuscript JH conception, critical review of manuscript from orthopedic point of view. All authors read and approved the final manuscript.
